# "Qualified Nurses" and Their Qualifications

**Published:** 1921-05-14

**Authors:** 


### THE MATRONS' AND SISTERS' DEPARTMENT.

### "QUALIFIED NURSES" AND THEIR QUALIFICATIONS.

The "Qualified Nurse" came into existence in July 1901 as the result of an Order of the Local Government Board of Ireland. It must be said of this Order, which created something of a sensation, that it initiated a bold scheme of nursing reform. The condition of Irish workhouse infirmaries prior to 1901 may be dimly guessed at from the report of the Privy Council on a petition addressed to them by certain Guardians who felt themselves aggrieved by the measures taken to insure common care for the sick poor. Dr. Biggar, Inspector under the Board, stated before the Privy Council that there had been great difficulty in getting the Guardians to appoint efficient nurses. There were twenty-eight workhouse infirmaries in Ireland in which there was no trained nurse whatever. In the Armagh workhouse at an inquest held on the body of a patient it transpired that there was only one paid nurse for sixty patients and twenty-three female lunatics. At the Derry Union Infirmary, containing from fifty-five to sixty-five sick, the Guardians rejected the medical officer's recommendation of a trained nurse and appointed a farmer's daughter who had never been inside a ward. Contemporary records give a most painful, it may be said revolting, account of the treatment to which the unhappy inmates were subjected in various parts of Ireland.

The movement in favour of reform was seriously checked by the difficulty of finding trained nurses willing to undertake duty in Irish workhouses, and a corresponding difficulty in finding assistants. The Local Government Board Order solved the problem by (*a*) abolishing nursing by paupers, (*b*) insisting on the appointment of a trained nurse in every workhouse, and (*c*) providing for a class of assistants to be called "qualified nurses." Trained nurses must have resided for not less than two years in a general clinical or other hospital recognised by the Board and have obtained a certificate after examination. "Qualified nurses" were women who had "obtained a certificate of proficiency in nursing after examination from a public general hospital or a workhouse infirmary and fever hospital or a nursing institution recognised as an efficient school by the Board." No term of residence was prescribed. The certificate of proficiency could be obtained on the very easiest conditions from minor infirmaries, and from the first it was evident to all acquainted with the facts that the "qualified nurse" in practice amounted to any strong young woman of good character who could be got to undertake the work.

So great was the benefit expected from the introduction of a trained nurse into every workhouse infirmary that the ambiguous definition of the "qualified nurse" was suffered to pass. In England later on very strong exception was taken to the proposed introduction of the term. In Ireland the controversy centred round the "trained nurse," and only a few voices were uplifted to suggest that the "qualified nurse" might come in time to compete with nurses who had won their title to proficiency after paying a largish premium and devoting three years to learning their business.

It was known to all from the first that many of the institutions claiming to produce "qualified nurses" had no opportunities for teaching beyond what one overworked nurse could find time to give, that they had not sufficient variety of cases, nor of surgical treatment, and no adequate appliances to give training worthy of the name. Worst of all the general atmosphere of the workhouse wards was rarely such as would imbue the assistants with any sound standard of professional duty and manners.

The "qualified nurses" have now been in existence nearly twenty years, and there must be a considerable number of them. So far the fear of their competing with trained nurses does not appear to have been realised. But another evil has arisen, likely to have even worse consequences. The Irish authorities were undoubtedly handicapped as regards staff in reforming the system of workhouse nursing. Perhaps the best that could be done was to give a brief preparatory course to the large number of women required to carry out under trained nurses the reforms so urgently required. It was believed that these subordinates could not be obtained at low salaries without the bribe of calling them "qualified." And the slur cast on the profession at large by the use of this ill-advised term has hardly till the present moment been appreciated. How was it legally possible in forming a State register of nurses to turn down the claims of women who had been definitely labelled "qualified" by a Government department? To have increased their pay and found them another name would have been the proper course from the outset. It would certainly have saved trouble in the end.

